# Effect of Atmospheric-Pressure Cold Plasma on Pathogenic Oral Biofilms and *In Vitro* Reconstituted Oral Epithelium

**DOI:** 10.1371/journal.pone.0155427

**Published:** 2016-05-25

**Authors:** Juliana Aparecida Delben, Chaiene Evelin Zago, Natalia Tyhovych, Simone Duarte, Carlos Eduardo Vergani

**Affiliations:** 1 Department of Dental Materials and Prosthodontics, Araraquara Dental School, Sao Paulo State University, Araraquara, Sao Paulo, Brazil; 2 Department of Basic Science and Craniofacial Biology, College of Dentistry, New York University, New York, New York, United States of America; National University of Singapore, SINGAPORE

## Abstract

Considering the ability of atmospheric-pressure cold plasma (ACP) to disrupt the biofilm matrix and rupture cell structure, it can be an efficient tool against virulent oral biofilms. However, it is fundamental that ACP does not cause damage to oral tissue. So, this study evaluated (1) the antimicrobial effect of ACP on single- and dual-species biofilms of *Candida albicans* and *Staphylococcus aureus* as well as (2) the biological safety of ACP on *in vitro* reconstituted oral epithelium. Standardized cell suspensions of each microorganism were prepared for biofilm culture on acrylic resin discs at 37°C for 48 hours. The biofilms were submitted to ACP treatment at 10 mm of plasma tip-to-sample distance during 60 seconds. Positive controls were penicillin G and fluconazole for *S*. *aureus* and *C*. *albicans*, respectively. The biofilms were analyzed through counting of viable colonies, confocal laser scanning microscopy, scanning electron microscopy and fluorescence microscopy for detection of reactive oxygen species. The *in vitro* reconstituted oral epithelium was submitted to similar ACP treatment and analyzed through histology, cytotoxocity test (LDH release), viability test (MTT assay) and imunnohistochemistry (Ki67 expression). All plasma-treated biofilms presented significant log_10_ CFU/mL reduction, alteration in microorganism/biofilm morphology, and reduced viability in comparison to negative and positive controls. In addition, fluorescence microscopy revealed presence of reactive oxygen species in all plasma-treated biofilms. Low cytotoxicity and high viability were observed in oral epithelium of negative control and plasma group. Histology showed neither sign of necrosis nor significant alteration in plasma-treated epithelium. Ki67-positive cells revealed maintenance of cell proliferation in plasma-treated epithelium. Atmospheric-pressure cold plasma is a promissing approach to eliminate single- and dual-species biofilms of *C*. *albicans* and *S*. *aureus* without having toxic effects in oral epithelium.

## Introduction

Several studies have reported colonization of tooth, prostheses, restorative materials and implants by yeast and bacterial biofilms causing several related diseases [1]. Oral biofilm has also been associated with occurance of aspirative pneumonia, endocarditis and orther systemic disorders [2]. Biofilms represent an aggregation of microbes embedded within a protective carbohydrate matrix, which allows adhesion to a host surface, stabilization of biofilm architecture, maintenance of nutrients, and protection against antimicrobial agents and host immune response [3, 4]. In this sense, *Candida albicans* is a frequent fungal biofilm-forming pathogen that causes life-threatening infections by colonizing medical and dental devices (i.e. prostheses, implants and catheters) [5, 6]. Dimorphism is an important characteristic experienced by *C*. *albicans* in response to adverse environmental conditions, which actually increases its virulence. Furthermore, coaggregation between *C*. *albicans* and oral bacteria has been reported as an important factor in microbial colonization and progression of infections in oral cavity [7]. Accordingly, *Staphylococcus aureus* is an oral bacteria that may cause local infections (e.g. endodontic problem, mucositis, angular cheilits, and parotitis) [8] and systemic diseases (e.g. endocarditis, pneumonia, eczema, abscesses, and septicemia) [9]. Adhesion of *S*. *aureus* to *C*. *albicans* hyphae has been also demonstrated [10], which increases its virulence and ability to tissue invasion. A previous study showed that dentures are commonly colonised by an association of yeast and bacteria, including *C*. *albicans* and *S*. *aureus*, even in healthy subjects [11]. Literature has also suggested that these pathogens can be commonly associated as co-infector microorganisms and the clinical outcome results are increased frequency and severity of the disease [10, 12, 13]. Thus, the dual-species biofilm of *C*. *albicans* and *S*. *aureus* should be further investigated due to its high pathogenicity [14] and resistance to antimicrobial agents [13, 15].

Current treatment of biofilm-related diseases involves mechanical removal of biofilm and the use of antiseptics and antibiotics. Nevertheless, considering that mechanical removal could be ineffective on biofilm inactivation, and that pathogenic microorganisms may develop resistance to antimicrobial agents, alternative therapies are required. In this sense, atmospheric-pressure cold plasma (ACP) is a promising tool against *C*. *albicans* [6, 16], *S*. *aureus* [17–19], and other microorganisms [20–22] that could be used for clinical treatment of biofilm-related oral diseases. The cold plasma is generated by gas ionization at atmospheric pressure and low temperature; producing reactive oxygen species (ROS), reactive nitrogen species (RNS), UV radiation, ions, electrons, excited molecules, and electromagnetic field [18, 23, 24]. Although plasma effect on microorganism is not fully understood, it has been demonstrated that the reactive species cause cell damage and/or death through dehydration, membrane lysis [19], and damage to DNA, lipids and proteins [25, 26]. It is also noteworthy that plasma plume can reach sites inaccessible by other methods (i.e. deep and interproximal caries and periodontal pockets), so it could be more effective for biofilm decontamination than conventional therapies.

Considering that plasma is usually produced by low-toxicity gases and its activity involves a mixture of products that decay within a few seconds, this approach has been suggested as environmentally friendly with no harmful residues [17]. Thus, production of stable plasma at atmospheric pressure has attracted attention for treating living human cells and tissues without thermal damage [27]. However, studies of the biological safety of plasma are limited [28, 29], particularly on oral mucosa [22]. Given that a novel treatment for biofilm-related oral diseases should not cause irreversible damage to the host cells, further studies are required to evaluate the biocompatibility of plasma on oral tissues.

The aim of this study was to evaluate (1) the antimicrobial efficacy of ACP on single- and dual-species biofilms of *C*. *albicans* and *S*. *aureus*, and (2) the biological safety of ACP on *in vitro* reconstituted oral epithelium.

## Materials and Methods

### Atmospheric-pressure cold plasma (ACP)

The ACP was generated through ionization of argon gas (Ar) at atmospheric pressure using the device Kinpen (INP, Germany) developed by Leibniz Institute for Plasma Science and Technolog*y* (INP) [30]. The device consists of a hand-held unit (170 mm in length, 20 mm in diameter and 170 g) for generation of plasma jet, a dc power supply (system power: 8 W at 220 V, 50/60 Hz), and a gas supply unit. The atmospheric-pressure plasma jet was generated from the top of the centered electrode and expanded to the surrounding air outside the nozzle [6]. The treatment of biofilm and oral epithelium samples was conducted at plasma tip-to-sample distance of 10 mm during 60 seconds in continuous working mode [30]. The samples were moved horizontally during plasma application for scanning of the overall surface. The plasma tip-to-sample distance was maintained using an apparatus developed to fix plasma positioning during treatment. The experiment was conducted by a calibrated operator (JAD) that was trained to perform the experiment in a standardized manner in order to minimize bias. The argon gas flow was set to 5 slm and the flow rate was controlled by a flow controller (MKS Instruments, Germany).

### Single- and dual-species biofilms

Strains of *C*. *albicans* (ATCC SC5314) and *S*. *aureus* (ATCC 25923) were used for *in vitro* biofilm culture [12]. Stock cultures were maintained at—80°C, streaked onto specific medium (Sabouraud Dextrose Agar with chloramphenicol for *C*. *albicans* and Mannitol Salt Agar for *S*. *aureus*) and incubated at 37°C for 48 hours. Each microorganism culture was transferred to 10 mL of specific medium (Yeast Nitrogen Base—YNB with 100 mM of glucose for *C*. *albicans* and Tryptic Soy Broth—TSB for *S*. *aureus*) and incubated at 37°C for 18 hours. Cells of each microorganism culture were harvested, washed twice with 10 mL of phosphate-buffered saline (PBS—10 mM PO_4_^3-^, 137 mM NaCl, 2.7 mM KCl) at 4000 rpm for 5 minutes, resuspended in 10 mL of RPMI medium (*Roswell Park Memorial Institute*) (RPMI-1640, Sigma-Aldrich Co., USA), and standardized to 1 X 10^7^ cells/mL. For single-specie biofilm (*C*. *albicans* or *S*. *aureus)*, 1 mL of the standardized cell suspension was added to 1 mL of RPMI medium, while for dual-species biofilm (*C*. *albicans + S*. *aureus*), 1.0 mL of the standardized cell suspension of each microorganism were mixed.

The biofilms were cultured on heat-polymerized acrylic resin discs (Vipi Wave, VIPI Ind. e Com. Exp. e Imp. de Produtos Odontológicos Ltda., Brazil) with 10 mm in diameter and 2 mm in thickness. The surface roughness of the discs was standardized to Ra = 3.0 μm [31] using a specific matrix. The discs were sterilized using microwave energy for 3 minutes at 650W in sterile ultrapure water [32].

Initially, the samples were incubated in the inoculum for 90 minutes [33] at 37°C in an orbital shaker. Then, non-adherent cells were removed by gently washing twice with 2 mL of PBS, and 2 mL of fresh RPMI medium was added to each sample. After 24 hours of incubation at 37°C in an orbital shaker, 1 mL of the RPMI medium was removed and equal volume of fresh medium was added for additional 24 hours of incubation under similar conditions [34]. After 48-hour incubation [12], a mature biofilm was formed so each disc was transferred to a new well and the biofilm was gently washed with 2 mL of PBS.

Experimental samples were submitted to ACP treatment, whereas negative control had no treatment. For positive control, *C*. *albicans* and *S*. *aureus* strains were exposed to fluconazole (80mg/L) and penicillin G (100mg/L) for 30 minutes at 37°C, respectively.

### *In vitro* reconstituted oral epithelium (ROE)

The *in vitro* reconstituted oral epithelium EpiOral^TM^ (ORL-200, MatTek Corporation, USA) was used in this study. The EpiOral^TM^ model is based on primary oral keratinocytes cultured on a collagen-based matrix [35].

Upon receipt, the cell culture inserts were maintained in pre-warmed MatTek assay medium into a humidified 37°C, 5% CO_2_ incubator for 1 hour to recover from stress of shipping. Following 1 hour incubation, medium was aspirated and replaced by pre-warmed fresh assay medium.

Similar to biofilm samples, experimental tissues were submitted to ACP treatment and the negative control had no treatment. Experimental and negative tissues were maintained into a humidified 37°C and 5% CO_2_ incubator for 24 hours after the experiments. Positive control tissues were incubated in similar conditions, except that 400 μL of 1% Triton X-100 solution (MatTek Corporation, USA) was added atop the tissue samples at 20 hours of incubation.

### Biofilm analyses

For counting of viable colony forming units (CFU), the biofilm was removed from the surface of the resin discs using sterilized cotton swab, which was subsequently transferred to a 1.5 mL centrifuge tube with 1 mL of PBS. After vortexing, serially diluted aliquots were plated to determine the number of CFU/mL of each microorganism after 24 hours of incubation at 37°C. *C*. *albicans* colonies were cultured in Sabouraud Dextrose Agar supplemented with chloramfenicol (0.1 mg/mL) [12] while *S*. *aureus* colonies were cultured in Brain Heart Infusion Agar supplemented with amphotericin B (0.025 mg/mL) [13]. The experiment was conducted in triplicate in three independent occasions. Data were analyzed by ANOVA and Tukey’s test (p<0.05).

The biofilm was also stained using the Live/Dead BacLight Viability kit (Invitrogen-Molecular Probes, USA) to reveal the proportion of live or active cells (fluorescent green) and dead or inactive cells (fluorescent red) [30]. The live/dead stain was prepared by diluting 1.5 μL of staining component A (SYTO 9) and 1.5 μL of staining component B (propidium iodide) in 1.0 mL of sterile 1% phosphate buffered solution (PBS–pH7.4) (Invitrogen, USA). Each sample was covered with 1 mL of the reagent mixture and incubated for 20 minutes at room temperature protected from light exposure. A series of images were obtained in the z section using a Leica TCS SP5 II confocal microscope (Leica, Germany) with the objective Leica HCX APO L 40x/0.8 W U-V-I water dipping lens (Leica, Germany). Five representative optical fields were examined for each specimen. The experiment was conducted in duplicate in two independent occasions.

For scanning electron microscopy (SEM), discs containing biofilm were fixed with 4% paraformaldehyde for 1 hour and then dehydrated in increasing ethanol concentrations (70%, 85%, and 100%) for 5 minutes in each solution. After dehydration, the samples were placed in a desiccator for 24 hours before gold sputter coating. The images were captured using the SEM Hitachi S-3500N (Hitachi High Technologies America, USA) under 8 mm working distance and 10–15 kV. The experiment was conducted in duplicate in three independent occasions.

The presence of intracellular reactive oxygen species (ROS) in plasma-treated biofilms was observed using the fluorescent probe dihydrorhodamine 123 (DHR 123) (Invitrogen-Molecular Probes, USA) [36–38]. This product is an uncharged and nonfluorescent ROS indicator that can passively diffuse across membranes where it is oxidized to cationic rhodamine 123, which exhibits green fluorescence. The biofilms were incubated with 15 μM DHR 30 minutes before ACP treatment. Fluorescent pictures were captured in the stereoscopic microscope Nikon SMZ1500 (Nikon Corporation, Japan) using 1.6 × lens and 5.0 × zoom with the microscope imaging software NIS-Elements Br (Nikon Corporation, Japan).

### ROE analyses

The CytoTox-ONE^TM^ Homogeneous Integrity Assay kit (Promega, USA) was used as a fluorometric method to estimate cell cytotoxicity based on the release of lactate dehydrogenase (LDH) from cells with damaged membrane [35, 39]. LDH release into the culture medium was measured with this enzymatic assay that results in the conversion of resazurin into resorufin. After 24-hour incubation, the culture medium of each sample was collected, transferred to 96-well plate (100 μL of culture medium per well, totalizing 6 measurements for each sample) and cooled to room temperature. Then, the kit reagent was added to each well (100 μL per well) and incubated for 10 minutes. After addition of stop solution (50 μL per well), fluorescent signal was measured in a microplate reader (SpectraMax M5 Multi-Mode microplate reader, Molecular Devices, USA) with excitation wavelength of 560 nm and emission wavelength of 590 nm. The experiment was conducted in duplicates in three independent occasions. Percent cytotoxicity was determined according to the following calculation:

$$\text{Percent cytotoxicity}=100\;\text{x}\;\frac{\left(\text{Experimental}–\text{Culture Medium Background}\right)}{\left(\text{Maximum LDH Release}–\text{Culture Medium Background}\right)}$$ (1)

The MatTek MTT toxicology kit (MTT-100, MatTek Corporation, USA) was used as a colorimetric assay to check tissue viability [40]. According to this method, the NAD(P)H-dependent cellular oxidoreductase enzymes present in viable cells are capable of reducing the tetrazolium dye MTT to its insoluble formazan, which has a purple color. The tissue inserts were gently rinsed twice with PBS and placed in 24-well plate containing 300 μL of MTT solution (MTT concentrate + MTT diluent) per well. After 3 hours of incubation at humidified 37°C and 5% CO_2_, the tissue inserts were transferred to another 24-well plate and immersed into 2 mL of the extractant solution per well. After 2 hours of extraction under agitation protected from light, the extractant solution was added into a 96-well plate (200 μL per well, totalizing 6 measurements for each sample) to determine the optical density at 570 nm in a microplate reader (SpectraMax M5 Multi-Mode microplate reader, Molecular Devices, USA). The extractant solution was used as a blank. The experiment was conducted in duplicates in three independent occasions. Percent viability was determined according to the following calculation:

Percent viability=100x[OD(sample)/OD(negative control)] (2)

Data about cytotoxity and viability were analyzed by ANOVA test with Dunnett’s or Tukey’s post-hoc for multiple comparison. p<0.05 was considered significance.

Histological analysis was conducted in order to study the microscopic anatomy of the cells and tissue architecture. For histological analysis [35], the samples were immersed in 10% formalin, washed with PBS and dehydrated with 50% and 70% ethanol. Samples were then prepared for paraffin embedding, cutting and hematoxylin and eosin staining. The slides were scanned using the digital slide scanner ScanScope CS2 (Aperio, USA) at 20X. The experiment was conducted in duplicate in two independent occasions.

To identify actively proliferating cells, immunohistochemistry was performed on 4 μm formalin fixed, paraffin embedded samples using a cell proliferation marker (rabbit anti-human Ki67 clone SP6) (Thermo Scientific, USA) [35]. The sections were deparaffinized in xylene, rehydrated through graded alcohols (100% and 95% ethanol) and rinsed in distilled water. Heat induced epitope retrieval was performed in a 1200-Watt microwave oven at 100% power in 10 mM sodium citrate buffer, pH 6 for 20 and 10 minutes, respectively. Sections were allowed to cool for 30 minutes and then rinsed in distilled water. Antibody incubation and detection were carried out at 40°C on a Discovery instrument (Ventana Medical Systems, USA) using Ventana’s reagent buffer and detection kits. Endogenous peroxidase activity was blocked with hydrogen peroxide. Antibody was diluted (1:400) in Dulbecco’s Phosphate Buffered Saline, (Life Technologies, USA). Samples were incubated overnight at room temperature. Antibody was detected with biotinylated goat anti-rabbit diluted 1:200 (Vector Laboratories, USA) for 30 minutes. This was followed by application of streptavidin-horseradish-peroxidase conjugate. The complex was visualized with 3,3 diaminobenzidene and enhanced with copper sulfate. Slides were washed in distilled water, counterstained with hematoxylin, dehydrated and mounted with permanent media. The experiment was conducted in duplicate in two independent occasions.

## Results

### Biofilms

**Fig 1:**
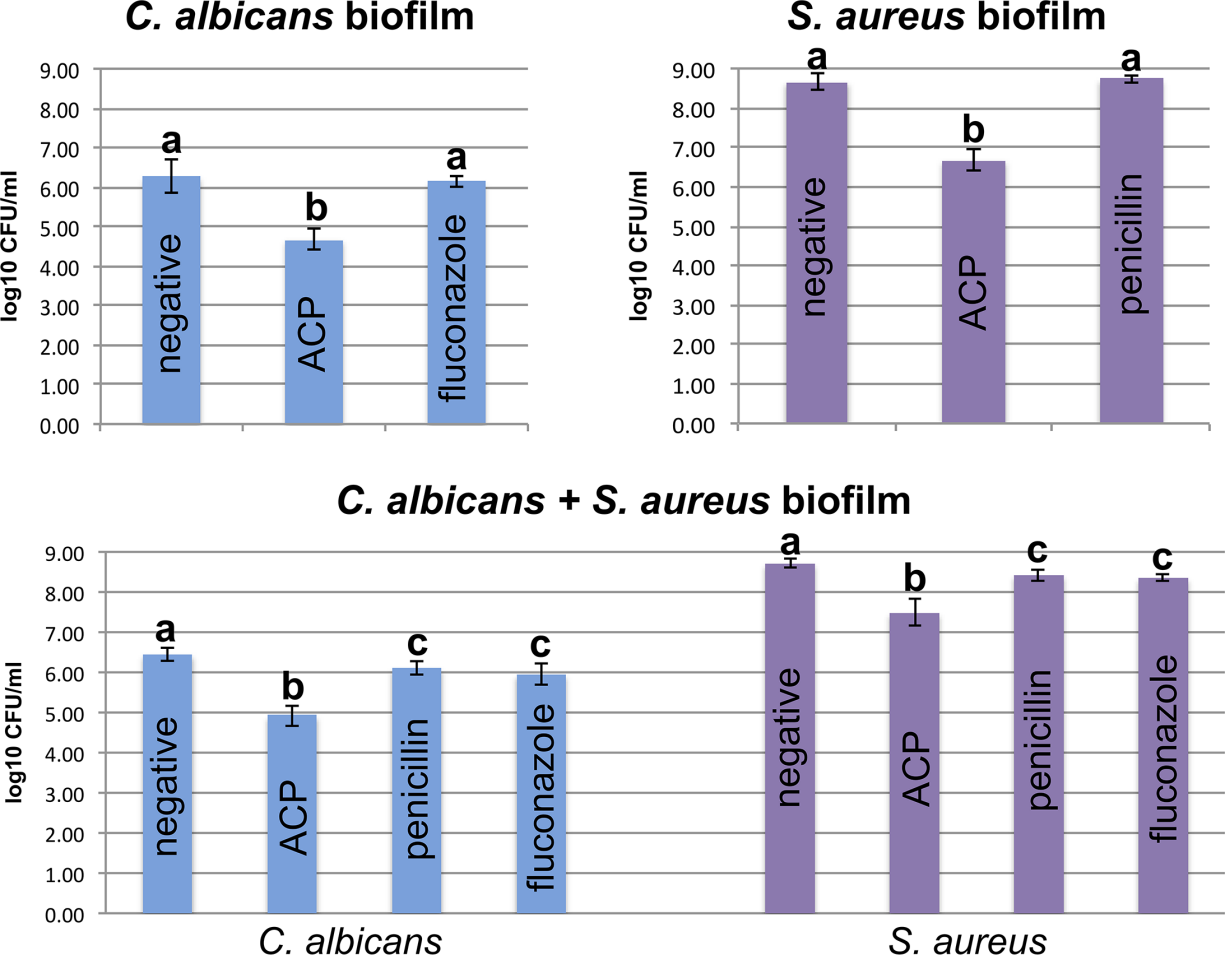
Log_10_ CFU/mL for both *C*. *albicans* and *S*. *aureus* strains in single- and dual-species biofilms. **Individual statistical analyses were conducted only for comparison between the negative control group, ACP group and positive control group (i.e. penicillin for *S*. *aureus* and fluconazole for *C*. *albicans*) of the same strain in each biofilm.** * different letters denote statistically significant difference (p<0.05) only between groups of the same strain in each graph.

Fig 1 shows the results of log_10_ CFU/mL of *C*. *albicans* and *S*. *aureus* for single and dual-species biofilms. It was observed a significant log_10_ CFU/mL reduction in all plasma-treated samples in comparison to the negative and positive controls. For single-specie biofilms, plasma group showed lower log_10_ CFU/mL (*C*. *albicans*: 4.68 ± 0.25 / *S*. *aureus*: 6.68 ± 0.29) than negative (*C*. *albicans*: 6.27 ± 0.42 / *S*. *aureus*: 8.67 ± 0.21) (p<0.05) and positive (*C*. *albicans*: 6.14 ± 0.12 / *S*. *aureus*: 8.74 ± 0.10) (p<0.05) controls. However, there was no significant difference in log_10_ CFU/mL between the negative and positive control samples (p>0.05). In dual-species biofilm, for both *C*. *albicans* and *S*. *aureus* strains, plasma group showed lower log_10_ CFU/mL (*C*. *albicans*: 4.92 ± 0.25 / *S*. *aureus*: 7.48 ± 0.33) than negative (*C*. *albicans*: 6.44 ± 0.16 / *S*. *aureus*: 8.71 ± 0.12) (p<0.05) and positive (*C*. *albicans*—fluconazole: 5.94 ± 0.25 / *C*. *albicans*—penicillin: 6.10 ± 0.18 / *S*. *aureus*—fluconazole: 8.35 ± 0.08 / *S*. *aureus*—penicillin: 8.42 ± 0.14) (p<0.05) controls. Positive controls also showed lower log_10_ CFU/mL than negative control (p<0.05). However, there was no significant difference (p>0.05) between positive controls with fluconazole and penicillin (Fig 1).

**Fig 2:**
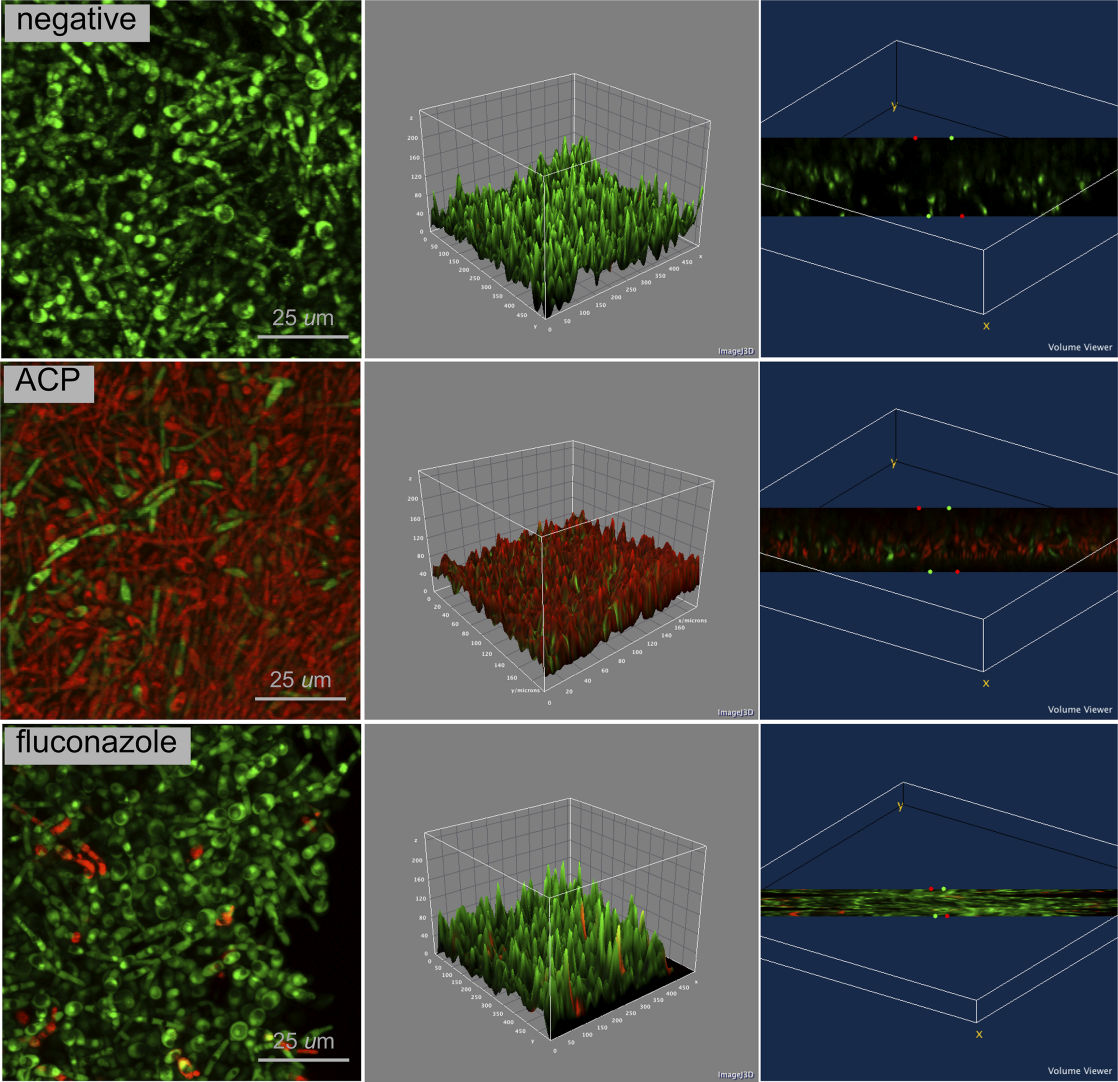
Confocal laser scanning microscopy of *C*. *albicans* biofilm in negative control group, ACP group and positive control group (fluconazole); 4 × zoom. Biofilm stained with Live/Dead BacLight Viability kit. Live cells in fluorescent green and dead cells in fluorescent red. 2D view (left side image), surface plot of 3D volume image (center image), and cross-section of 3D volume image (right side image) show the distribution of live and dead cells throughout biofilm layers.

**Fig 3:**
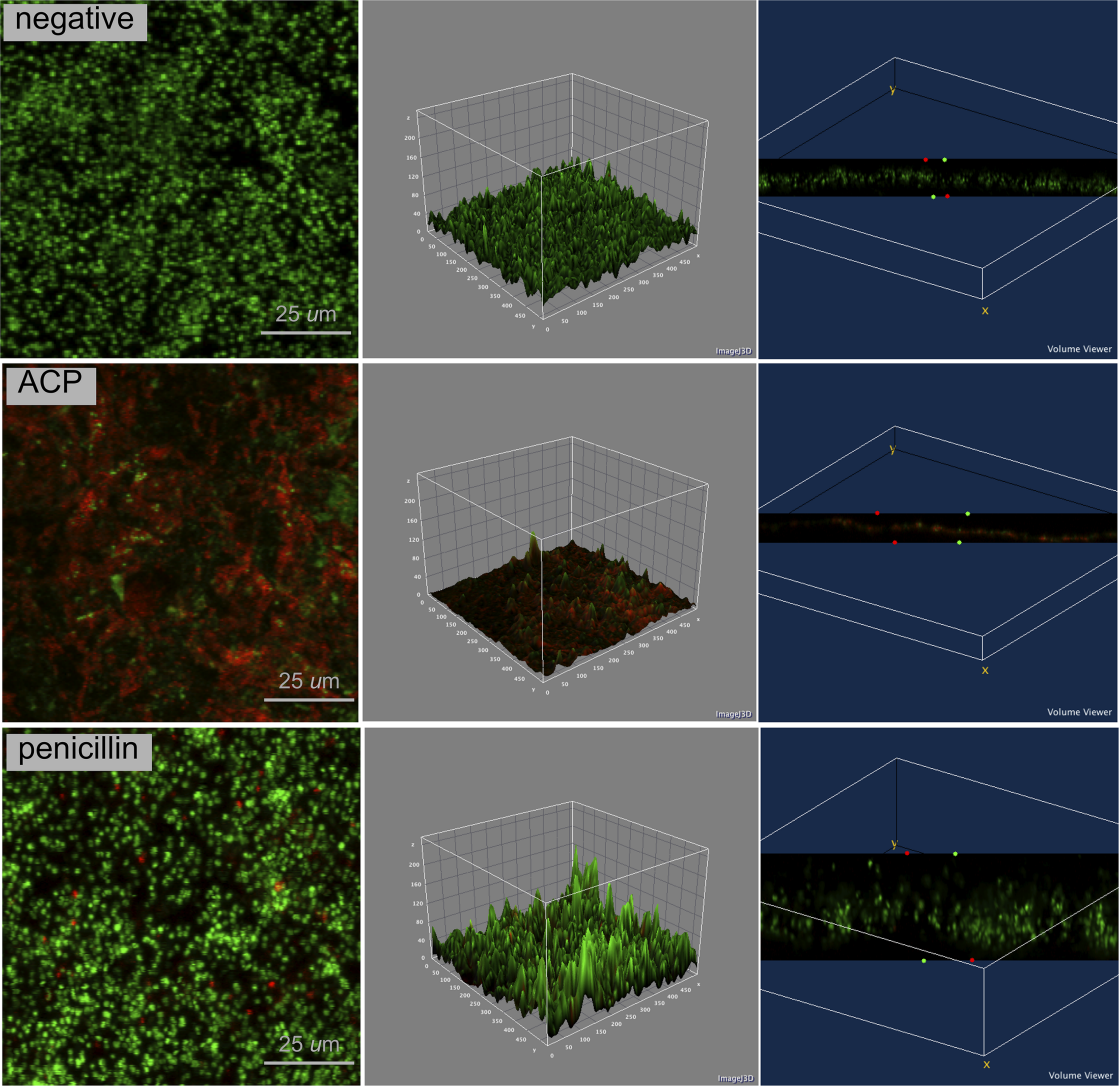
Confocal laser scanning microscopy of *S*. *aureus* biofilm in negative control group, ACP group and positive control group (penicillin); 4 × zoom. Biofilm stained with Live/Dead BacLight Viability kit. Live cells in fluorescent green and dead cells in fluorescent red. 2D view (left side image), surface plot of 3D volume image (center image), and cross-section of 3D volume image (right side image) show the distribution of live and dead cells throughout biofilm layers.

**Fig 4:**
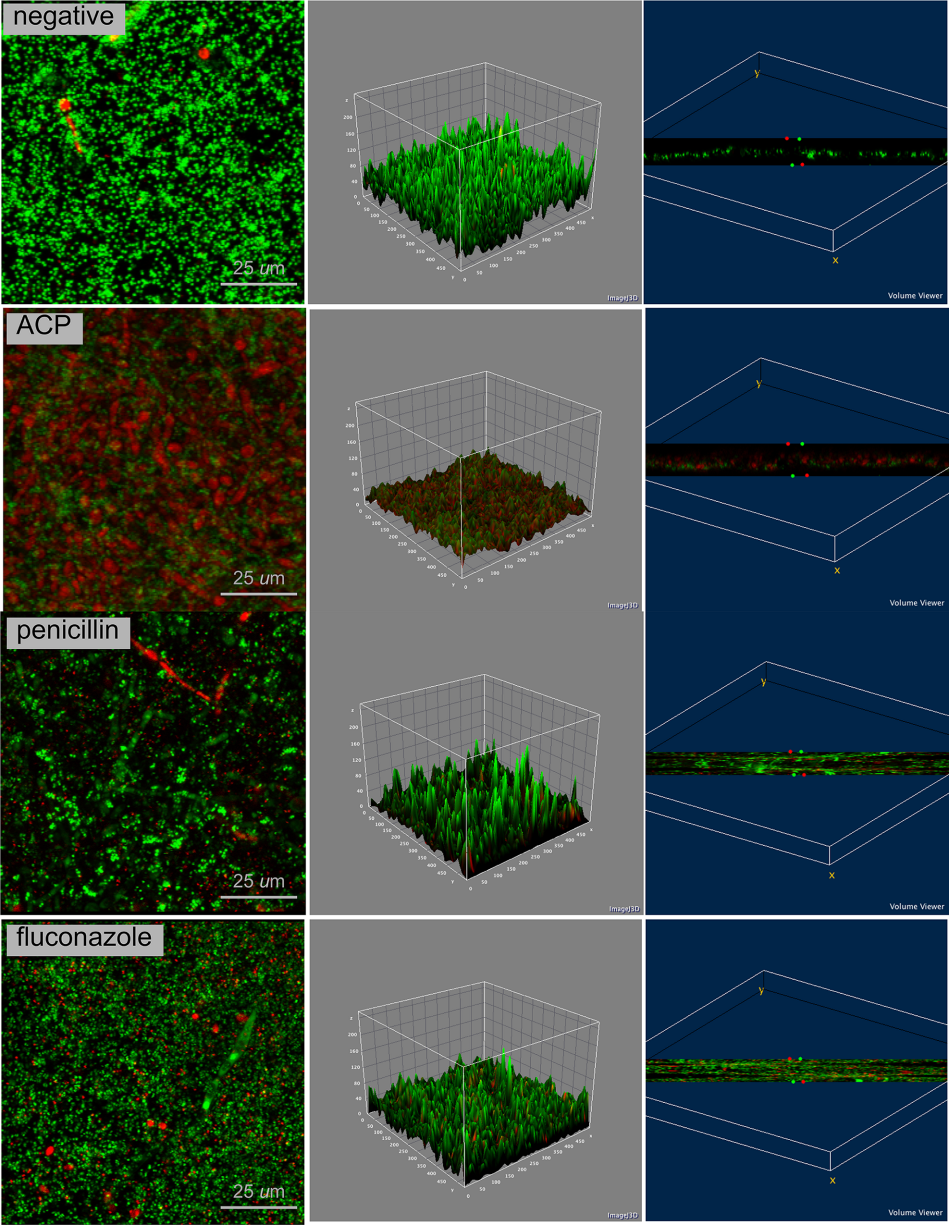
Confocal laser scanning microscopy of *C*. *albicans* + *S*. *aureus* biofilm in negative control group, ACP group and positive control groups (fluconazole and penicillin); 4 × zoom. Biofilm stained with Live/Dead BacLight Viability kit. Live cells in fluorescent green and dead cells in fluorescent red. 2D view (left side image), surface plot of 3D volume image (center image), and cross-section of 3D volume image (right side image) show the distribution of live and dead cells throughout biofilm layers.

Confocal analysis (Figs 2–4) clearly demonstrates that plasma-treated samples showed higher amount of dead biofilm than the negative and positive control groups. As displayed by the 3D volume images, dead cells were seen even at bottom layer of samples treated with plasma. After plasma treatment, images suggest higher survival of *S*. *aureus* when this microorganism was grown in a dual-species biofilm model compared with the single-specie biofim.

**Fig 5:**
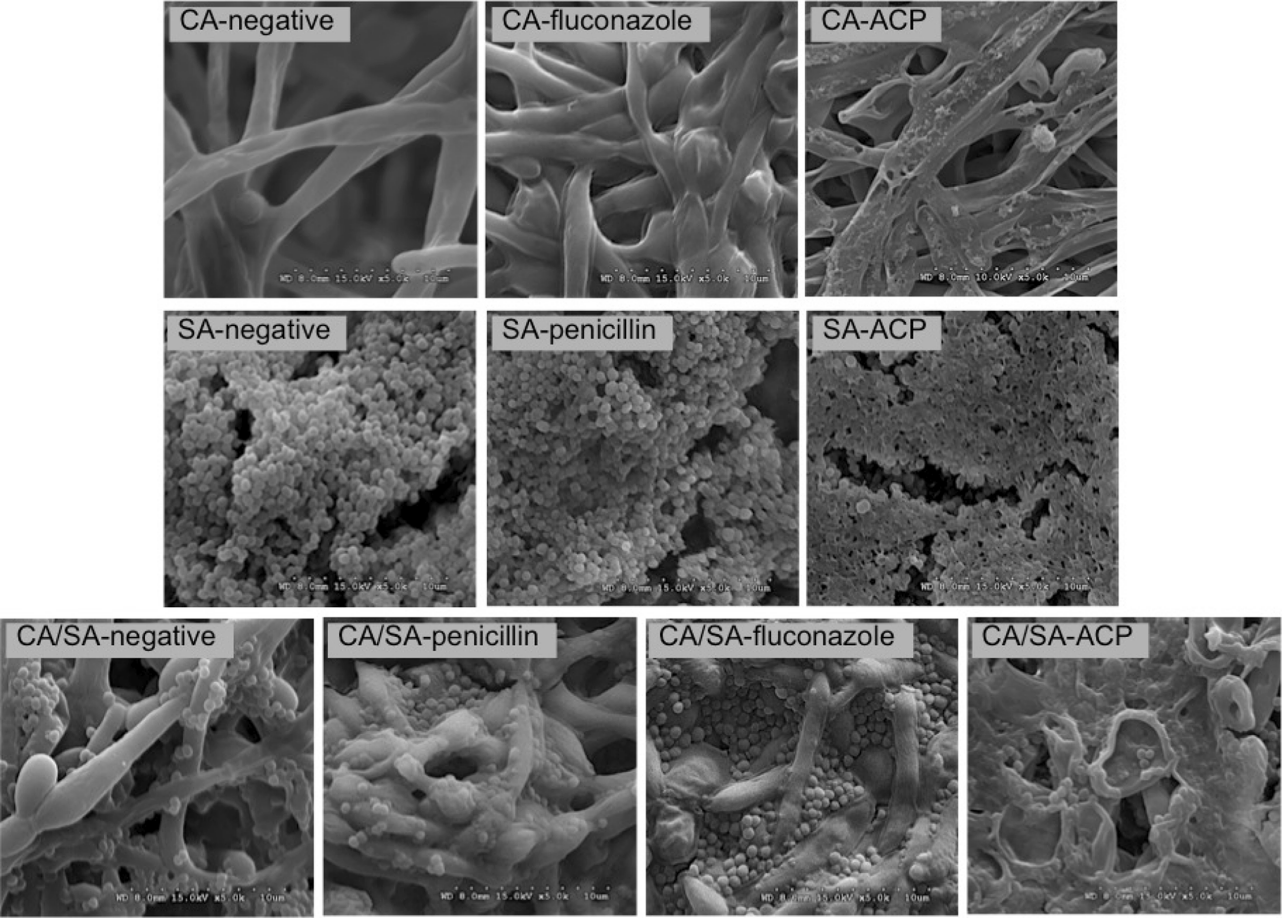
SEM micrographs of single and dual-species biofilms in negative control group, ACP group and positive control groups (fluconazole and penicillin); 5000 × zoom. *C*. *albicans* (CA), *S*. *aureus* (SA) and *C*. *albicans* + *S*. *aureus* (CA/SA) biofilms. Note rupture of cells architecture in all biofilms after ACP treatment (groups CA-ACP, SA-ACP and CA/SA-ACP).

All plasma-treated biofilms showed alteration in the morphological structure of the microorganisms/biofilm in comparison to the negative and positive controls. SEM micrographs (Fig 5) revealed massive perforation of *C*. *albicans* and *S*. *aureus* cell wall and membrane after plasma treatment. As a consequence, plasma-treated biofilms seemed flatten, representing damage to biofilm architecture. In contrast, control samples looked as undamaged. In this sense, it was also observed adhesion of *S*. *aureus* to *C*. *albicans* hyphae in dual-specie biofilm, indicating some collaborative process between both microorganisms for host tissue invasion. Interestingly, it was found that some *S*. *aureus* cells remained preserved within *C*. *albicans* scaffold in the dual-species biofilm.

**Fig 6:**
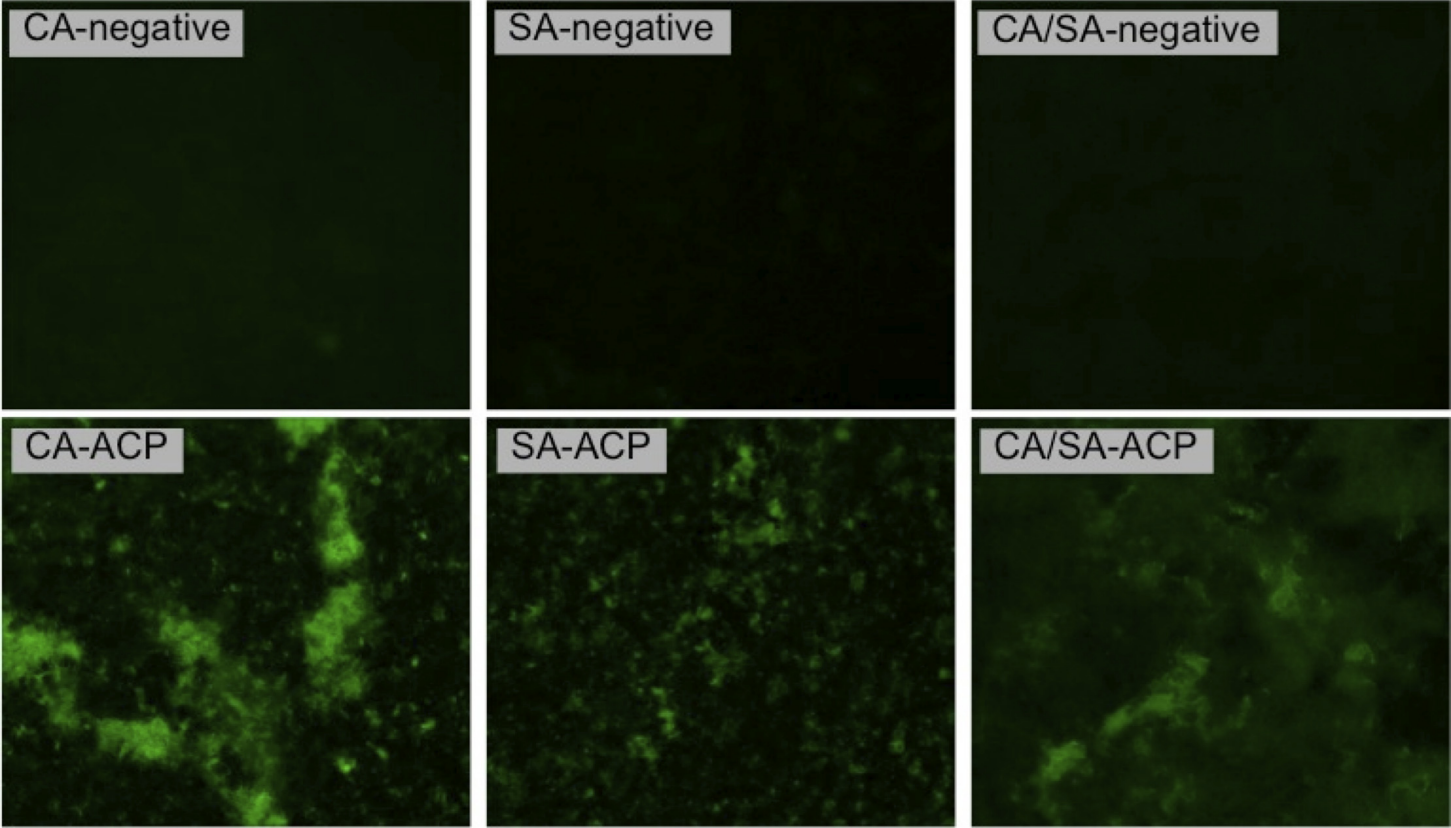
Fluorescence microscopy images of single and dual-species biofilms in negative control group and ACP group; 1.6 × lens and 5.0 × zoom. Fluorescent green (oxidized rhodamine 123) represents presence of intracellular ROS. CA—*C*. *albicans* biofilm, SA—*S*. *aureus* biofilm, and CA/SA—*C*. *albicans* + *S*. *aureus* biofilm.

Fig 6 demonstrates presence of ROS exclusively in plasma-treated biofilms, as shown by the fluorescent green (oxidized rhodamine 123). The detection of ROS in plasma-treated biofilms suggested some antimicrobial pathway of the atmospheric-pressure cold plasma used in this study.

### *In vitro* reconstituted oral epithelium (ROE)

**Fig 7:**
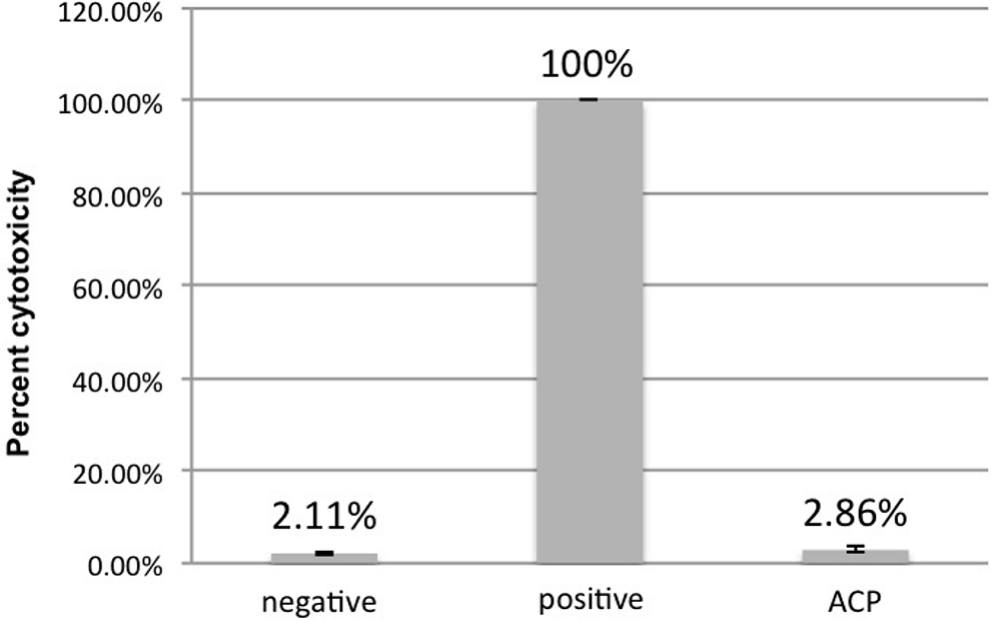
Mean (± SD) percent cytotoxicity values of ROE for negative control group, positive control group and ACP group.

The measurement of LDH release demonstrated lower percent cytotoxicity for negative control (2.11% ± 0.26) and plasma group (2.86% ± 0.56) in comparison to the positive control (100% ± 0.00). Considering the positive control as a reference for cytotoxicity calculation, the post-hoc Dunnett’s test showed significant difference between positive control and plasma group (p<0.05) as well as between positive and negative controls (p<0.05). In addition, Tukey’s test showed no significant difference between negative control and plasma group (p = 0.094) (Fig 7). The similarity between the results of the negative control and plasma samples indicates a non-cytotoxic effect of plasma treatment on oral epithelium within the dosage applied in this study.

**Fig 8:**
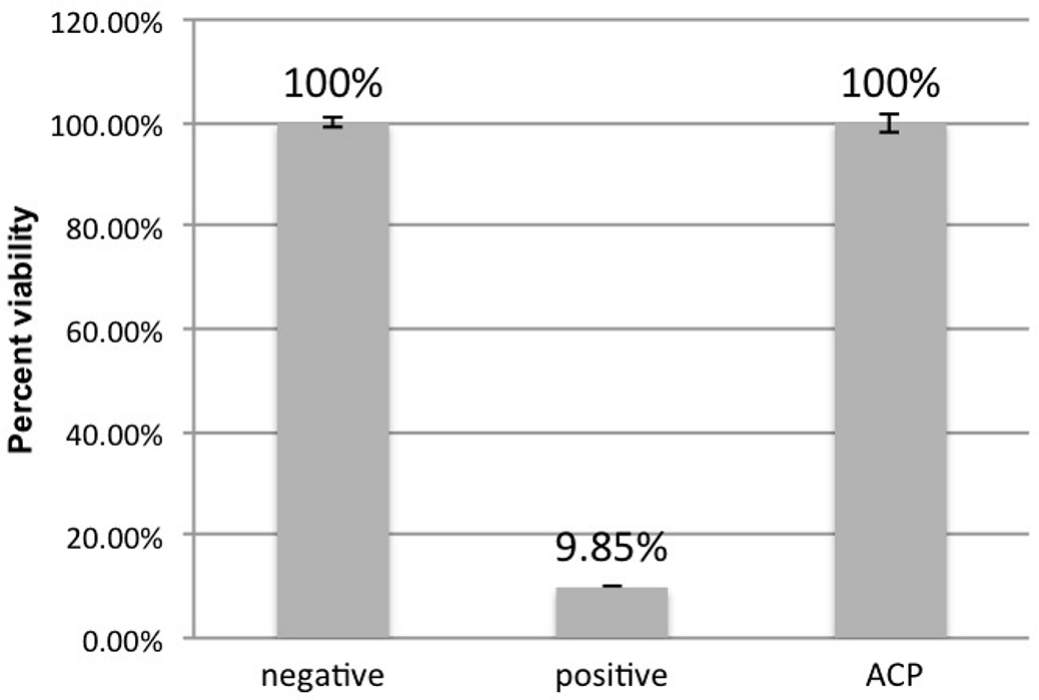
Mean (± SD) percent viability values of ROE for negative control group, positive control group and ACP group.

The MTT assay revealed high percent viability for both negative control (100% ± 0.00) and plasma group (100% ± 1.85) in comparison to the positive control (9.85% ± 0.84). Considering the negative control as a reference for viability calculation, the post-hoc Dunnett’s test showed no significant difference between negative control and plasma group (p = 0.596). However it was found a statistically significant difference (p<0.05) between positive and negative controls (Fig 8). The similarity between the results of the negative control and plasma samples corroborates with the cytotoxicity results.

**Fig 9:**
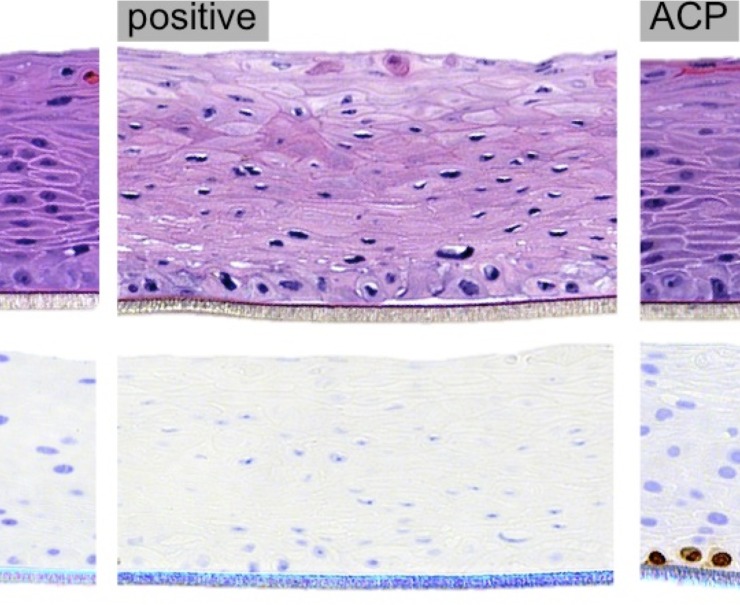
Oral epithelium sections of negative control group, positive control group and ACP group; 20 × zoom. Sections stained by hematoxylin and eosin for histology (top) and immunohistochemistry for Ki67 (bottom).

Histological analysis showed minor epithelium alteration in plasma samples with slight keratinization at the top layer in comparison to the negative control. In contrast, positive control exhibited significant tissue damage with cell vacuolization and nuclear shrinkage (i.e. pyknosis). Furthermore, Ki67-positive cells were found in the basal layer of plasma samples, representing actively proliferating cells, analogous to the normal epithelium of the negative control group. In contrast, positive control showed no Ki67 expression (Fig 9).

## Discussion

Cold plasma has emerged as a physical treatment with microbicidal effectiveness on bacteria, parasites, fungi, spores, and viruses [41]. In the present study, log_10_ CFU data (Fig 1) and confocal laser scanning microscopy (Figs 2–4) confirmed the antimicrobial effect of ACP since all plasma-treated samples exhibited significant lower viability than positive and negative controls. In addition, reactive oxygen species were found in all plasma-treated samples (Fig 6).

It has been suggested that the reactive oxygen species (i.e. ozone, atomic oxygen, superoxide, peroxide, hydroxyl radical, and nitric oxide) produced by plasma generate oxidative effects in cellular biomacromolecules including DNA, lipids and proteins [17]. As a consequence, oxidative stress causes lipid peroxidation and oxidation of several amino acids of proteins, which compromises the function and integrity of membrane and cell wall [6, 20]. In addition, membrane desestabilization affects the ability to maintain proper intracellular pH [4] and releases cellular contents in the surrounding medium [28]. It was also suggested that plasma species break down hydrogen, sulphide and peptide bonds of the proteins; leading to changes in protein structure and dramatically decrease of enzyme activity [42]. On the other hand, low doses of oxidative stress have been demonstrated to favor biofilm formation of some strains so that treatment time and dose must be carefully controlled [42].

The assumption about membrane damage caused by plasma was confirmed by SEM images (Fig 5), revealing cell rupture. Membrane perforation was previously reported since plasma reactive particles produce a general mechanical effect on the surface of living organisms called ‘etching’ [25]. Etching results from the reaction of highly reactive radicals with organic materials, generating by-products that are desorbed from the surface, causing membrane perforation [18]. In addition, charged particles generated by plasma may lead to membrane lysis when electrostatic force overcomes the tensile strength of the membrane [23].

The inactivation of biological agents promoted by ACP may also result from deconstruction of the microorganism genetic material (DNA) by UV radiation produced with plasma and erosion of the microorganisms through intrinsic photodesorption. The photon-induced desorption results from damage of chemical bonds in the microorganism after being exposed to UV radiation, allowing its atoms to form volatile compounds [25, 43]. However, the role of UV radiation in atmospheric-pressure plasma sterilization remains controversial [23]. According to some authors [43], even when no significant UV emission is present with low-temperature atmospheric-pressure plasma, the synergy of other species such as radicals and charged particles still plays a dominant role for sterilization.

In contrast to conventional therapy, literature suggests that a great benefit on using ACP is that antimicrobial resistance is less likely to occur because of its multiple modes of action and diversity of active agents [17, 42]. While strongly charged or chemically reactive agents in conventional therapy fail to reach the biofilm due to the negatively charged extracellular polymeric matrix, which acts as an ion-exchange barrier, some small molecules of plasma are able to travel into the biofilm by channels with varying diameters [19]. Interestingly, it was shown that pre-treatment with attenuated gas plasma restored antibiotic sensitivity of methicillin-resistant *S*. *aureus* (MRSA) [44]. In contrast, the results of positive control groups for both species showed that the drugs were not strongly effective to reduce biofilm viability probably because only a single dose was applied and not enough to inactivate the pathogens.

The results about reconstituted oral epithelium showed no sign of significant damage after ACP application, as demonstrated by the low cytotoxicity (Fig 7) and high viability (Fig 8) levels in plasma-treated samples. Only few studies have been conducted on the effect of ACP on eukaryotic cells [28]. In this sense, some authors [22] evaluated the effect of ACP treatment on buccal and tongue mucosa of rabbits. Even though the treatment was conducted for a much longer period (10 minutes) than that from the present study (one minute), the histological analysis showed no hyperemia, swelling, ulcer or anabrosis. Furthermore, the authors concluded that ACP might offer such advantages over the traditional methods as high bacterial killing efficiency, time saving and less toxic by-products produced. Similarly, the histological analysis in our study showed no sign of necrosis or significant alteration in oral tissue after ACP application (Fig 9). A previous study also reported no morphological changes or signs of necrosis or apoptosis while treating bacterial loads on the pig skin with cold plasma [29]. In another investigation [27], a 2- to 5-minute cold plasma treatment substantially inactivated ocular pathogens without causing significant tissue and DNA damage. Together with our results, these findings support future application of ACP for disinfection of living tissue.

Considering that both *C*. *albicans* and ROE cells are eukaryotic, a question remains about plasma mechanism that inactivated *C*. *albicans* without causing tissue damage at the same dosage. It was previously suggested that killing of *C*. *albicans* by neutrophils is driven synergistically by combinatorial effects of oxidative plus cationic stress. In this sense, although *C*. *albicans* activates signaling pathways for detoxification of the oxidative stress and repair of damage caused by ROS, cations inhibit catalase enzyme activity; which leads to the hyper accumulation of intracellular ROS [45]. Thus, it can be indicated that plasma effect against *C*. *albicans* is driven by a combination of oxidative stress and cation fluxes as a result of reactive species and charged particles, similarly to neutrophils pathway. However, it has been indicated that the effect of ROS in mammalian cells is dose-dependent, ranging from cell proliferation to cytotoxic damage with apoptosis [46]. Furthermore, at least 40 genes products are involved in the adaptive response of mammalian cells to oxidative stress through antioxidant defense, damage removal or repair enzymes [47]. As an early defensive response, mitotic mammalian cells also enter into a transient growth-arrested state to protect DNA from injuries caused by oxidative stress [47].

The absence of harmful effect observed on ROE can be also explained by the following mechanisms: 1- the lifetime of the atoms is very short (only several milliseconds) and fail to cause injury to the normal tissue, 2—the role of the UV is weak and it cannot damage mucosa in such a short period of exposure time, 3—mammalian cells are protected from ROS by their different cell metabolism and better resistance mechanisms to external stress, and 4—prokaryotic cells have a much higher surface to volume ratio so that a lower plasma dose is required for inactivation [22, 48].

The immunohistochemistry revealed Ki67-positive cells in the plasma-treated samples (Fig 9), which indicates maintenance of actively cell proliferation in oral epithelium after ACP application. Considering that Ki67-positive cells were present at basal layer, it can be suggested that plasma was not able to achieve deeper tissue structure. Keratinized epithelium may also have functioned a defense barrier against plasma reactive species. In addition, cold plasma can produce different effects in mammalian cells that range from cell proliferation to apoptosis, depending on plasma dose [49]. In this sense, sub-lethal doses of plasma have been used to induce apoptosis in cancer cells but not necrosis [50]. A previous study also demonstrated that ACP was effectively employed in the treatment of chronic skin and wound infections by simultaneously killing bacteria and promoting wound healing [42].

It is also noteworthy that the relatively low temperature is a critical feature of ACP system for *in vivo* applications since the majority of the electrical energy used to electrify the gaseous environment goes into the production of energetic electrons, instead of heating the surrounding gas [4]. So, ACP can be used on sensitive surfaces without causing heating, electric shock or pain [42]. However, although the literature confirms that ACP has several beneficial applications in medicine, there have not been sufficient studies to comprehensively assess the potential side effects of the treatment.

As a limitation, even in well-defined plasma-generating conditions it is difficult to characterize plasma and resulting active species for each individual reaction. So, further understanding and standardization are required to control microbial response and avoid possible cytotoxic effects [42]. In addition, although no visibly damage was observed in oral epithelium, direct plasma effects on animal tissues and immune cells should be further investigated [18].
